# Layer dependence and gas molecule absorption property in MoS_2_ Schottky diode with asymmetric metal contacts

**DOI:** 10.1038/srep10440

**Published:** 2015-05-20

**Authors:** Hyong Seo Yoon, Hang-Eun Joe, Sun Jun Kim, Hee Sung Lee, Seongil Im, Byung-Kwon Min, Seong Chan Jun

**Affiliations:** 1 School of Mechanical Engineering, Yonsei University, 50 Yonsei-ro, Seodaemun-gu, Seoul 120-749, Korea; 2 Institute of Physics and Applied Physics, Yonsei University, 50 Yonsei-ro, Seodaemun-gu, Seoul 120-749, Korea

## Abstract

Surface potential measurement on atomically thin MoS_2_ flakes revealed the thickness dependence in Schottky barriers formed between high work function metal electrodes and MoS_2_ thin flakes. Schottky diode devices using mono- and multi- layer MoS_2_ channels were demonstrated by employing Ti and Pt contacts to form ohmic and Schottky junctions respectively. Characterization results indicated n-type behavior of the MoS_2_ thin flakes and the devices showed clear rectifying performance. We also observed the layer dependence in device characteristics and asymmetrically enhanced responses to NH_3_ and NO_2_ gases based on the metal work function and the Schottky barrier height change.

Two-dimensional (2D) nano-sheets that can be exfoliated from layered materials such as graphite, BN, and MoS_2_ have been of intense interest because of their unique electrical, mechanical and chemical properties^1^,^2^,^3^,^4^,^5^,^6^,^7^. Since the field effect characteristic of single layer graphene was demonstrated with extremely high carrier mobility^8^,^9^, interest in such 2D nanomaterials dramatically increased because they are considered as promising materials for advanced nanoelectronic applications. For the last decade, numerous efforts have been made to implement such unique nano-sheets in practical electronic systems. Various techniques for large-scale synthesis and integration of 2D materials were discussed including solution-based deposition using chemical exfoliation^10^,^11^,^12^ and chemical vapor deposition (CVD)^13^,^14^,^15^. Recent investigations reported a layered heterostructure of various 2D materials by using a direct growth technique, which may enable practical and advanced nanoelectronic devices^16^,^17^. Unlike graphene, which has a zero or very small energy band gap, single layer MoS_2_ has a direct energy band gap very similar in size to silicon; thus, it has been used as an active layer with a high on/off ratio and high carrier mobility in field effect transistor devices^4^. Furthermore, recent studies of photo-excitation and chemical exfoliation of MoS_2_ layers showed tunability of the energy band structure with the layer thickness and metallic transition due to atomic structure deformation^18^,^19^. Also, unlike bulk MoS_2_, which shows clear n-type behavior, some MoS2 thin film based transistor devices showed ambipolar characteristics depending on the surrounding environments (e.g., ionic liquid and polymer gate dielectric), and its carrier mobility for holes was even higher than that for electrons^20^,^21^. A scanning tunneling microscope (STM) measurement on a MoS_2_ nanosheet^22^ also demonstrated p-type semiconducting behavior, which depended on the surface defect concentration. These previous results strongly support the possibility of energy band structure engineering and suggest a wide range of applications of MoS_2_ in the field of electronics and optoelectronics.

To realize a MoS_2_ thin film based CMOS device that may possibly replace silicon-based technology and overcome its fundamental limitations, understanding physical phenomena at the interface between MoS_2_ and a metal electrode is very important. Since the Schottky barrier height in the metal/semiconductor contact is closely related to the difference between the work function of the metal and the electron affinity of the semiconductor, it has been predicted that metal electrodes with a low work function such as Ti would show better contact properties with a MoS_2_ thin film than a high work function metal electrode like Pt or Au. Based on this prediction, a previous study revealed that a MoS_2_ field effect device using Sc source and drain electrodes (work function <4 eV) showed superior performance compared to that using high work function metal electrodes; further, the effect of Fermi level pinning close to the conduction band of MoS_2_ was also reported^23^. However, a clear and detailed explanation of the physical phenomena occurring at the interface between MoS_2_ and the metal contact has not yet been given. In particular, the layer dependence on a MoS_2_/metal Schottky contact has never been reported.

In this study, we designed and fabricated a MoS_2_ thin film diode device with an asymmetric metal contact to explore Schottky barrier formation with a high work function metal. Ti electrodes and Pt electrodes were applied to a MoS_2_ thin flake to form ohmic contacts and Schottky contacts, respectively. The surface potential difference profile of the MoS_2_ layers and visualization of charge depletion around Pt electrodes using electrostatic force microscopy (EFM) demonstrated the Schottky contact formation and the layer dependence. The device was operated and analyzed under n-type behavior of the MoS_2_ channel. The source-drain current showed highly rectifying behavior based on the high Schottky barrier between the Pt electrode and single layer MoS_2_. However, multi-layer MoS_2_ showed a higher reverse current because of the relatively small Schottky barrier height. This result indicates that a band structure engineering is potentially possible for the hetero-junction device using two different 2D materials.

To evaluate the layer dependent energy band structure of MoS_2_, we employed EFM, which can provide the contact potential difference between a probe tip and sample from surface band bending^24^. When the conducting tip and sample are brought close enough, there will be electrostatic force due to the work function difference of the two materials as
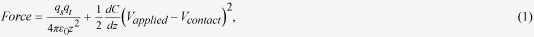
where *dC/dz* is the derivative of the sample-tip capacitance, *q*_*s*_ is surface charge, and *q*_*t*_ is charge induced on the tip. A voltage consisting of an AC/DC bias is applied between the tip and sample. The tip has a specific resonance frequency, *ω*, and the scanning V_DC_ (DC bias) is used to minimize the electrostatic force and eliminate the ω term; this DC bias represents the measured surface potential, V_contact_.

Figure 1(a) shows the image of MoS_2_ flake measured by AFM. We can see that the flake is consists of 4 different regions and the thickness for each region is represented in Fig. 1(c). Since the thickness of single layer MoS_2_ was estimated less than 1 nm in a previous study^4^, our result indicates 1-, 2-, 3- and 4-layer of MoS_2_ respectively. Figure 1(b) represents the surface potential result of the same flake. The normal distribution based on the surface potential result shows the layer dependence as shown in Fig. 1(d). This result is corresponding to a previous study showed the same measurement with epitaxially grown graphene layers and supports the layer dependent energy band gap change of MoS_2_^18^,^25^. According to the surface potential result, the thicker MoS_2_ layer seems to have the higher electron affinity and work function value since the potential value here represents the difference between the work function of the tip and the surface as below^26^,^27^,
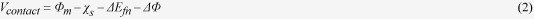
where *Ф*_*m*_ is the metal work function of the tip, *χ*_*s*_ is the electron affinity of the surface, *ΔE*_*fn*_ is the Fermi level position referenced to the bottom of the conduction band, and *ΔФ* represents band bending caused by surface states. If the surface state distribution is locally negligible, *V*_*contact*_ can represent the work function of target surface. A relatively larger difference in the potential value between 2 and 3 layers can be due to the band gap change or substrate effect since the single layer MoS_2_ can be more affected by surface charges on SiO_2_ substrate while the effect on 2 layers or thicker MoS_2_ can be screened by their bottom-most layer.

Figure 2(a) represents a schematic image of the energy band structure for the MoS_2_ layers and the Pt electrode based on the surface potential result and Fermi level shift. The strong interlayer coupling of electron orbitals on sulfur atoms and the quantum confinement effect of single layer MoS_2_ is the main reason for the transition of energy band structure when the thickness of MoS_2_ is reduced from bulk to single layer^28^,^29^,^30^. The result suggests a relatively lower Schottky barrier height at the interface between the thicker MoS_2_ and the high work function metal. In Fig. 2(b), we tested several devices with various flake thicknesses. The thinner MoS_2_ sample tends to have a lower current amplitude and relatively nonlinear I-V_DS_ characteristic which suggests a high contact resistance and Schottky barrier. Figure 2(c,d) show a more clear comparison in the current flow. To eliminate unintended fluctuations among the samples, we tested the current flow over three different areas having thickness of 2 nm, 5 nm and 20 nm respectively in a single flake as shown in Fig. 2(c). Figure 2(d) shows I-V_DS_ characteristics of the device. The current through the thickest layer (the current from electrode C1 to C2) showed a much higher amplitude compared to that through the thinnest layer (the current from electrode A1 to A2). Also, the thicker layer showed more symmetric and ohmic I-V_DS_ characteristics while the thinnest layer showed clear asymmetric and Schottky characteristics. Since the device was designed carefully by the electron beam lithography to have identical channel lengths and contact areas, the differences can be understood by differences in electron concentrations and the Schottky barrier height with its thickness. According to the energy difference between the conduction band of MoS_2_ and the work function of Pt electrode, the thicker layer of MoS_2_ can have the relatively lower Schottky barrier height.

For further analysis, we applied Ti/Pt asymmetric metal contacts on MoS2 device and made MoS2 Schottky diode devices. A schematic image of the device is illustrated in Fig. 3(a). Figure 3(b) shows an image of the device obtained by AFM. Thickness of the MoS_2_ flake was estimated as 5 nm and each metal electrode was designed to have the same distance from the adjacent electrodes to eliminate the effect of channel length. Figure 3(d) shows the device image measured by EFM, which provides the surface potential difference for Pt and Ti contacts, respectively. The relative difference in surface potential can be observed by the change in color, and that for MoS_2_ is in between that of the Ti and Pt electrode. We can also confirm the layer-dependent change. The thicker part of the MoS_2_ flake showed a lower *V*_*contact*_.

In the EFM result for the device, the potential difference between Ti and MoS_2_ was very small. On the other hand, the area that is covered by a Pt electrode shows a large potential difference compared to MoS_2_ and the Ti electrode as shown in Fig. 3(c), a normal distribution of the measured surface potential in each area. Based on the small difference in the potential between the Ti electrode and MoS_2_, we assumed that the effect of Schottky barrier height at the Ti/MoS_2_ interface on the device characteristics is small enough to be ignored and we considered Pt/MoS_2_ Schottky contact only^23^.

A schematic of the energy band structure of Schottky diode device is illustrated in Fig. 4(a) based on the surface potential measurement. The actual value by the EFM measurement was a little higher than the work function of Ti, which is around 4.2 eV. This value can be slightly varied due to the surface states of MoS_2_ and defects. Figure 4(b) shows corresponding Schottky diode characteristics in the proposed energy band structure. Since the Ti/MoS_2_ junction shows a very small Schottky barrier height and almost ohmic contact, the device with asymmetric contact will show rectifying operation based on the Schottky barrier at the MoS_2_/Pt junction. With these asymmetric metal contacts, the source-drain current showed a much higher level under the forward bias voltage (+ for Pt drain electrode and – for Ti drain electrode) than in the case of symmetric Pt contacts. Under the reverse bias voltage, the source-drain current is almost the same as that of the Pt symmetric contacts, which has a very high Schottky barrier. This result supports the assumption that MoS_2_ forms ohmic contact with Ti, and the bottleneck of current flow in the device is the Schottky barrier with a Pt contact.

In Fig. 4(c), we observe that the source-drain current under reverse bias is almost the same as that under forward bias when a high positive back gate voltage is applied. This tendency can be explained by the Schottky barrier height modulation by the back gate bias, and it also suggests a Schottky transistor behavior of MoS_2_ thin film channel. A majority charge carrier concentration change in MoS_2_ thin flake is the main reason for the current level change with the gate bias. However, with the high positive gate bias, the Schottky junction can allow the high reverse current flow since a charge carrier injection under the reverse bias can be increased substantially as the barrier height is lowered by the gate bias. On the other hand, under the negative gate bias, the current gap between the forward and reverse bias increases because the reverse current is easily blocked, while the current flow under the forward bias is difficult to be turned off. Therefore, under the reverse bias, contrary to the forward bias, we observe a very low ‘off’ state current and a higher on/off ratio. The on/off ratio of the device during transistor performance is relatively small for the forward bias (<10^2^) compared to the reverse bias case (>10^4^), as shown in Fig. 4(b). The effect of the Schottky barrier on transistor performance can be shown more clearly when we compare the device with Ti/MoS_2_/Ti junctions (ohmic) and Pt/MoS_2_/Ti junctions (Schottky) as shown in Fig. 4(d). Under the reverse bias between the source and drain electrode, the current can be turned off with a relatively higher gate voltage since the Schottky barrier at Pt/MoS_2_ junction can effectively block the charge carrier injection from MoS_2_ as compared to the ohmic device, where the charge carrier concentration change due to the electric field effect is fully responsible for the transistor operation.

Under the assumption that the device has a Schottky contact between the Pt electrode and MoS_2_ flake, we can draw an energy band diagram, as shown in Fig. 5(a), with a Schottky barrier for the majority charge carrier according to the Fermi level pinning of MoS_2_ close to its conduction band with the high work function metal. Here, Фm is the metal work function and ФB is the Schottky barrier height. Red lines indicate the energy band of multi-layer MoS_2_ based on the assumption that the thicker MoS_2_ layer has higher electron affinity. Figures 5(b) represents Schottky diode performance of mono-layer MoS_2_ under various back gate biases with Ti and Pt as the source and drain electrodes, respectively. Under forward bias (V_SD_ > 0), the device exhibits a drastic current increase, which gradually increases with the back gate bias. With a +10 V back gate bias, the device showed the highest current amplitude. When a reverse bias (V_SD_ < 0) is applied to the device, the current is blocked by the high Schottky barrier. The ratio of the current under the forward bias and reverse bias is almost 104, and it seems to be changed by back gate bias modulation. On the other hand, with ten layer MoS_2_ device, the current ratio between the forward and reverse bias is less than 10 as shown in Fig. 5(c). According to the previous results, the Schottky barrier between the high work function metal and multi-layer MoS_2_ may be smaller than that of mono-layer MoS_2_. Therefore, the current under reverse bias cannot completely be blocked by the Schottky barrier compared to the mono-layer deivce and the I-V_DS_ characteristic shows less asymmetric feature. This layer dependent reverse current supports the surface potential difference of MoS_2_ layers and its effects on the device performance. Also, these two different device performances can provide significant advantage in gas molecule detection.

Absorption of gas molecules may also confirm the Schottky barrier effect on the device performance; in addition, we can be able to achieve much higher sensitivity for certain gases. In the device with mono-layer MoS_2_, the electrical resistance decreased when exposed to 10 ppm of NH_3_, as shown in Fig. 6(a). The device shows diode characteristics because of the Schottky barrier and an asymmetric current change between the forward and reverse bias. Under a forward bias, there is a slight change in the current during the gas reaction. Since NH_3_ is well known as an electron-donating molecule for 2D materials, the resistance decrease in the forward bias can be understood as the effect of charge carrier concentration change. However, under a reverse bias, the current, which is originally blocked by the Schottky barrier, showed a more drastic increase compared to the forward bias case. With the multi-layer MoS_2_ channel, there is a significant reverse current as shown in Fig. 5(c) and this relatively high reverse current, which is due to the smaller Schottky barrier, is drastically reduced when the device is exposure to 10 ppm of NO_2_ as shown in Fig. 6(b).

This behavior can be understood by the metal work function change as a result of gas molecule absorption. Previous studies showed the work function of Pt can be decreased by NH_3_ and increased by NO_2_^31^. Therefore, in the mono-layer MoS_2_ device, the Schottky barrier height at the Pt/MoS_2_ junction can be reduced to allow more reverse current under NH_3_ exposure and the opposite reaction can be occurred in the multi-layer MoS_2_ device under NO_2_ exposure. The signal change in reverse current is much greater than that in forward current. This difference confirms that the Schottky barrier formation and its modulation under ambient gas condition. Also, we observe the clear layer dependence in the reverse current change. The distinct differences from the layer thickness and bias direction support the result of surface potential measurement and confirm its effect on diode device.

This result can be a great advantage for improving gas response when we compare the response to NH_3_ under reverse bias (Fig. 7(a)) and forward bias (Fig. 7(b)) since the current under reverse bias is much lower than that of a forward bias in the pristine state. The work function of Ti also can be changed by gas molecule absorption. However, this effect seems much smaller than that for Pt since it already forms an ohmic contact with the MoS_2_. The response of the device to NH_3_ confirms the previous device performance originates from Schottky barrier formation at the Pt/MoS_2_ junction and its modulation based on the electric field effect.

In this paper, we showed the layer dependence of Schottky barrier height at the MoS_2_ **/** Pt interface and demonstrated a MoS_2_ Schottky diode device. By applying the asymmetric metal contacts, we observed clear current rectification performance with a MoS_2_ thin flake channel. The surface potential difference profile measured from EFM indicated a high Schottky barrier formation with Pt and ohmic contact formation with Ti. The device performance also showed results consistent with the suggested energy band structure. The gate voltage dependence in the current rectification performance and transistor characteristic indicates Schottky barrier height modulation by the electric field effect and highlights the importance of proper selection of a metal contact for the high performance MoS_2_ electronic devices. In the future, a clear and detailed explanation of a wide range of energy band structure deformation of MoS_2_ nanosheets including p-type transitions or ambipolar characteristics should be made since these highly tunable electronic properties can encourage the realization of practical and diverse applications in nanoelectronics.

## Method

### Fabrication of MoS_2_ devices

MoS_2_ flakes were prepared on highly doped Si/SiO_2_ substrates by mechanical exfoliation of bulk MoS_2_ purchased from SPI Supplies. Thickness of MoS_2_ was estimated via optical color contrast and confirmed by atomic force microscopy (Nanoscope IV, Veeco). 100 nm of Ti electrodes were patterned by electron beam lithography and deposited by electron beam evaporation followed by lift-off in acetone. The same processes were applied for Pt electrodes after Ti electrodes formation. The device performance was characterized by typical source/drain and source/gate voltage modulation (Keithley 2400 source meter) with room temperature probe station.

### Surface potential measurement

Interleave mode of electric force microscopy (Nanoscope IV, Veeco) with PtIr-coated n-doped silicon probe tip (SCM-PIT, Veeco) were used for surface potential measurements. Topological profile of the surface was provided by the first scan of the tip followed by its second scan to measure electrostatic force between the surface and tip. *V*_*contact*_ can be obtained by this technique as a feedback potential that minimize the amplitude of oscillation.

## Author Contributions

H.S.Y. wrote the manuscript and analyzed data. H.E.J. and B.K.L. performed surface potential measurement. S.J.K. prepared MoS_2_ samples. H.S.L. and S.I. provided useful advices and comments. S.C.J. supervised the work.

## Additional Information

**How to cite this article**: Yoon, H. S. *et al.* Layer dependence and gas molecule absorption property in MoS_2_ Schottky diode with asymmetric metal contacts. *Sci. Rep.* **5**, 10440; doi: 10.1038/srep10440 (2015).

## Figures and Tables

**Figure 1:**
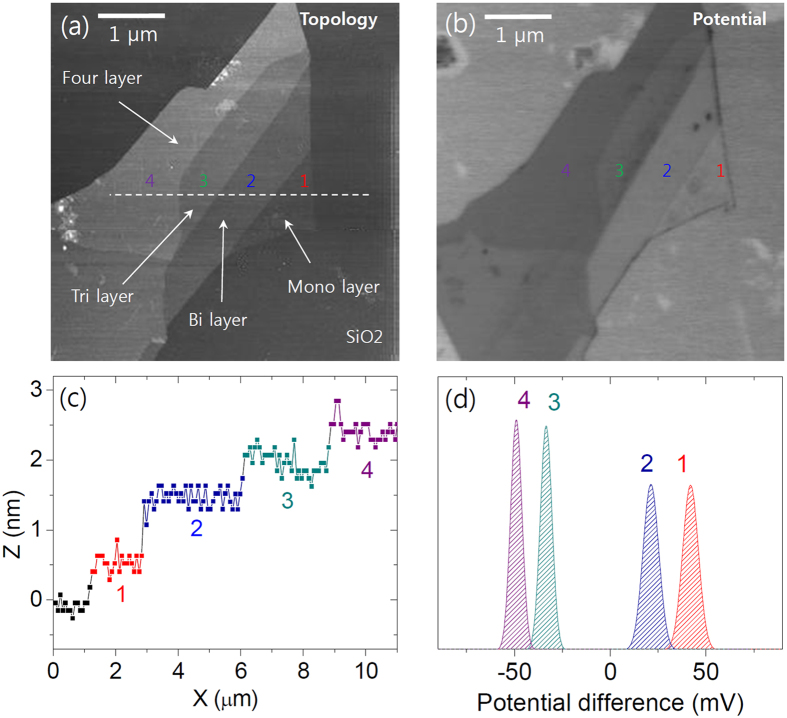
(**a**) MoS_2_ flake image measured by AFM and (**b**) surface potential measurement result of the same flake. (**c**) The thickness of flake is varied from 0.6 nm to 2.4 nm which are corresponding to 1 to 4 layers of MoS_2_. (**d**) Normal distribution of the relative surface potential difference for each layer.

**Figure 2:**
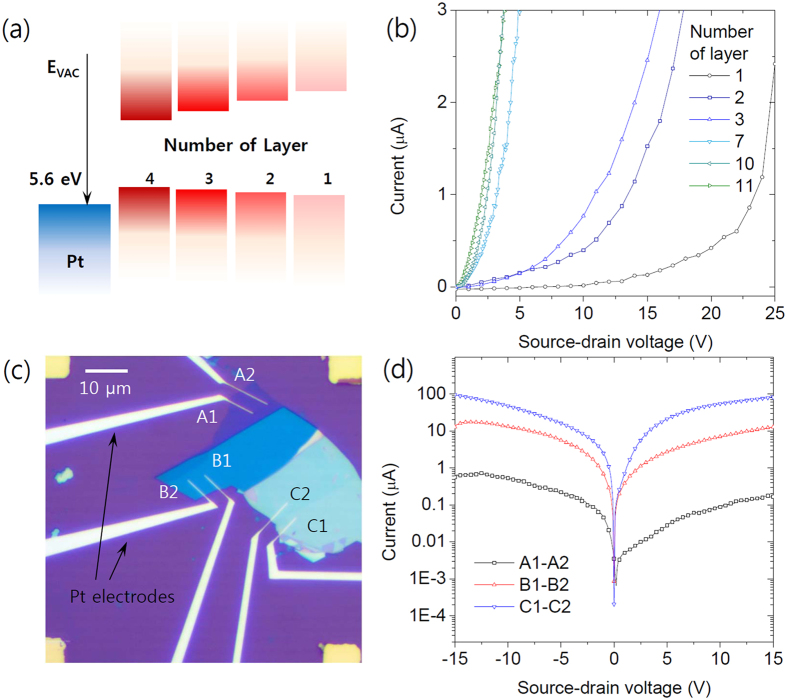
(**a**) Schematic image of the energy band alignment for MoS_2_ layers and Pt electrode according to the surface potential measurement. (**b**) I-V_DS_ characteristics of several MoS_2_ layers with different thicknesses. (**c**) Optical image of the device with Pt electrodes. (**d**) I-V_DS_ characteristics of the corresponding MoS_2_ flake. Thicker layer shows more ohmic characteristics.

**Figure 3:**
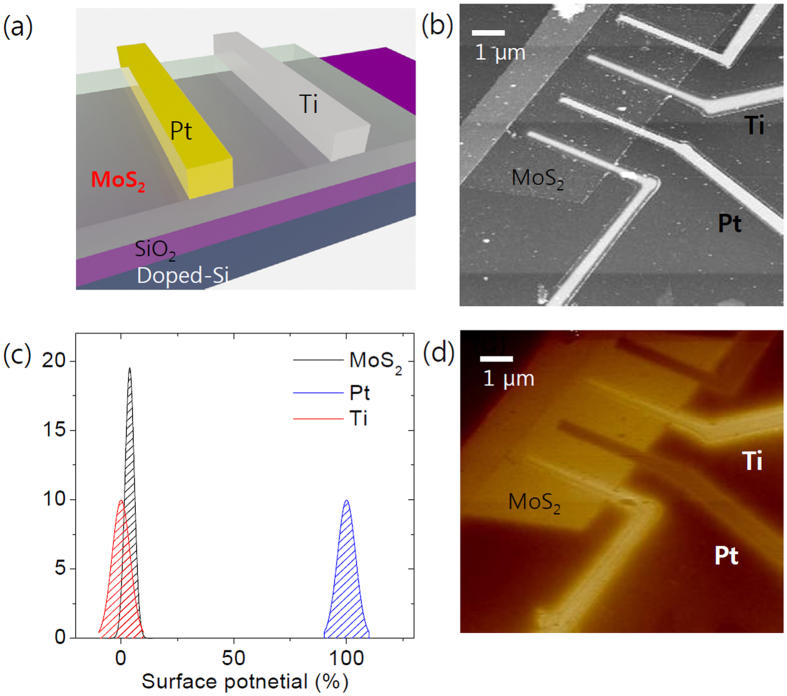
(**a**) Schematic view of MoS_2_ schottky diode device. Two pairs Ti and Pt electrodes were used to form ohmic and Schottky contacts with MoS_2_, respectively. (**b**) AFM image of the device. (**c**) Normal distribution of the relative work function differences for Ti, Pt and MoS_2_. (**d**) Surface potential measurement result of the same device.

**Figure 4:**
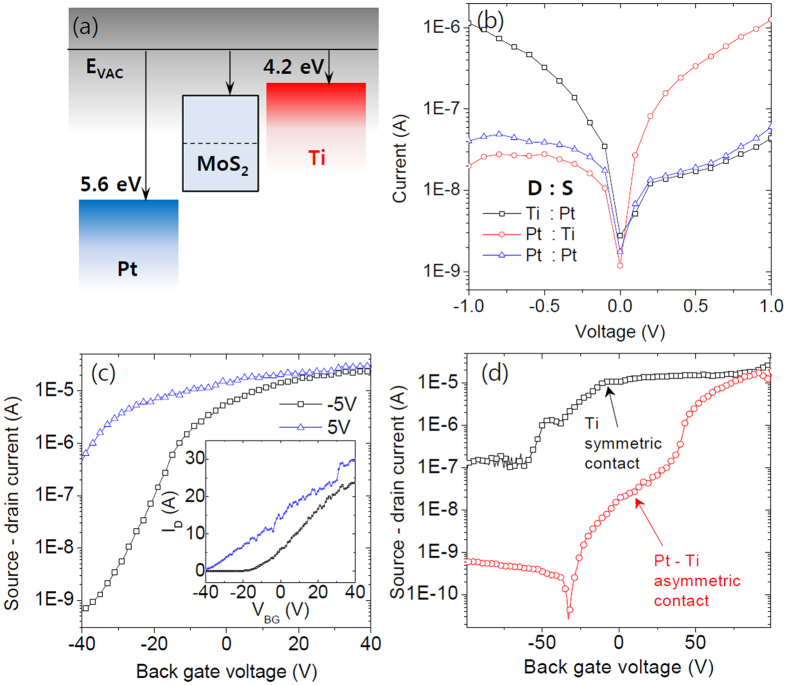
(**a**) Schematic image of the energy band alignment corresponding to the surface potential measurement. (**b**) I-V_DS_ characteristics for the Pt - Ti asymmetric contacts and Pt - Pt symmetric contacts. The asymmetric contact device showed clear diode performance. (**c**) Transfer curves for the device. The current amplitude is higher under the forward bias condition. However on/off ratio is much higher under the reverse bias due to its low ‘off’ state current. (**d**) Field effect transistor performance of two different types of device. Filed effect mobility is about 6 cm^2^/Vs for Ti symmetric contact device and 2 cm^2^/Vs for Ti/Pt asymmetric contact device. The device with asymmetric contact showed a much lower ‘off’ current compared to symmetric contact device.

**Figure 5:**
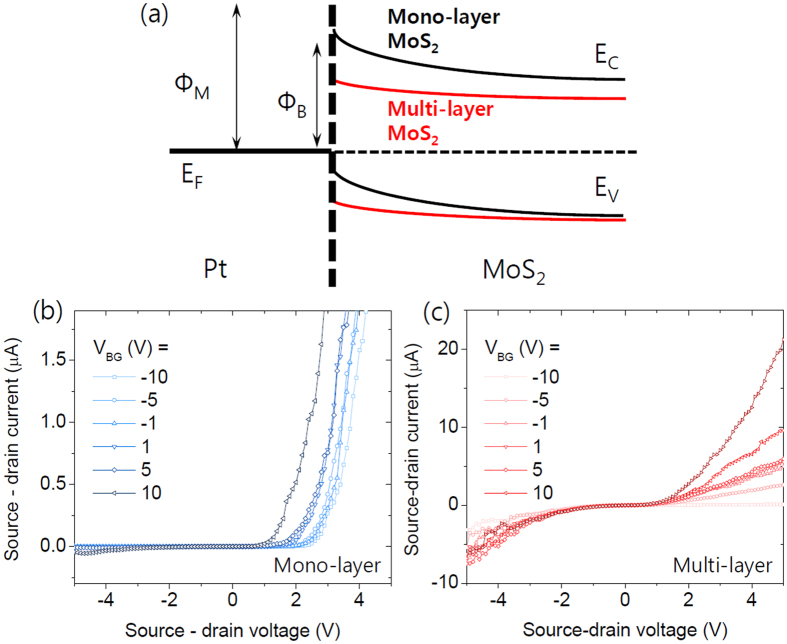
(**a**) Energy band diagram for the schottky junction at the Pt/MoS_2_ interface. The red curve represents the energy band ofr multi-layer MoS_2_. (**b**) I-V_DS_ characteristics for the Pt - Ti asymmetric contacts device with mono-layer MoS_2_ and (**c**) with multi-layer MoS_2_ (t = 10 nm).

**Figure 6:**
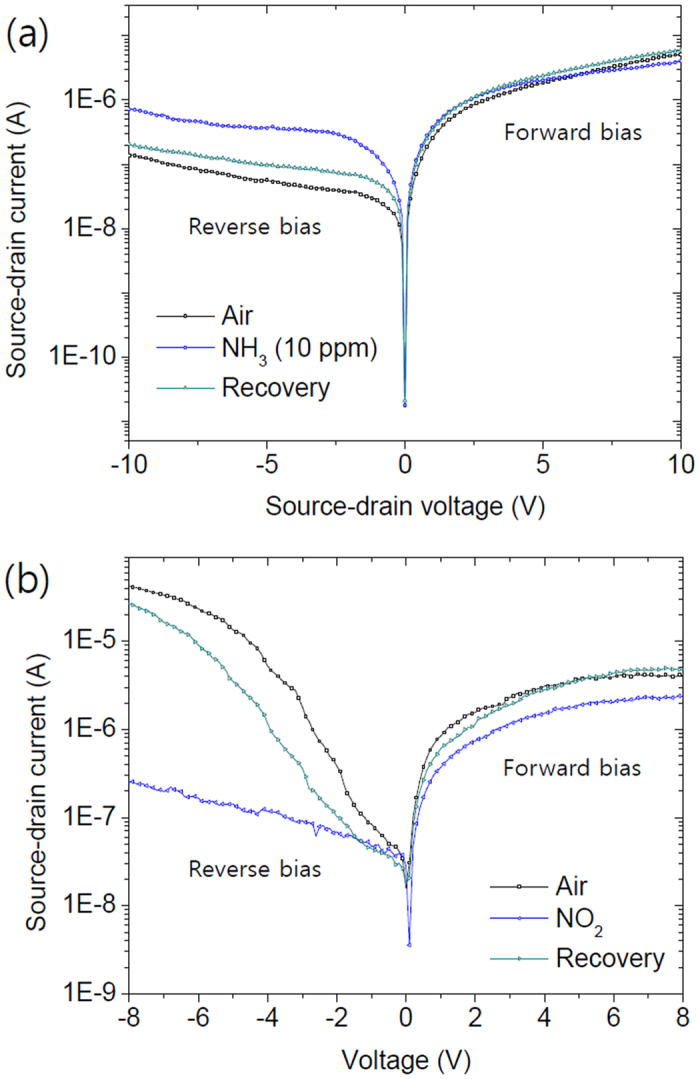
(**a**) I-V_DS_ characteristics of the asymmetric contact device with mono-layer MoS_2_ under NH_3_ exposure. (**b**) Result of same measurement with multi-layer MoS_2_ under NO_2_ exposure.

**Figure 7:**
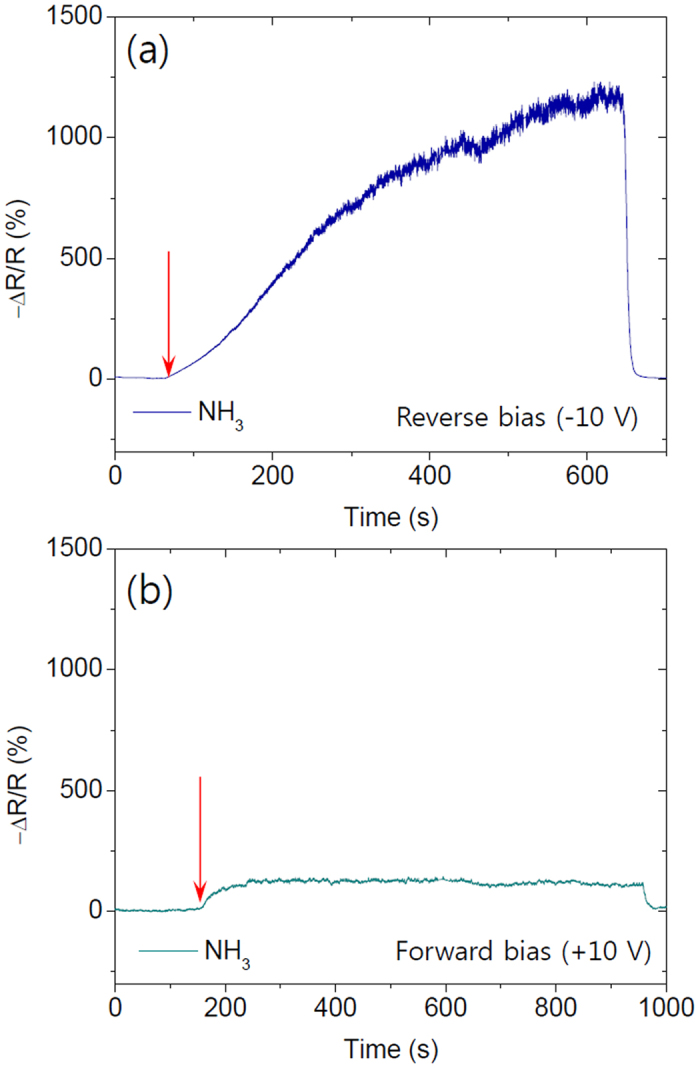
(**a**) Resistance change of the mono-layer device with reverse bias (−10 V) and (**b**) forward bias (+10 V) under 10 ppm of NH_3_ exposure.
